# Nanostructures Formed by Brass Electrochemical Oxidation—Fabrication Strategies and Emerging Applications

**DOI:** 10.3390/ma18081728

**Published:** 2025-04-10

**Authors:** Wojciech Jan Anioł, Piotr Dobroń, Katarzyna Tomczyk, Wojciech J. Stępniowski

**Affiliations:** Institute of Materials Science & Engineering, Military University of Technology, Kaliskiego 2 Str., 00908 Warsaw, Poland; u64594@student.wat.edu.pl (W.J.A.); piotr.dobron@student.wat.edu.pl (P.D.); katarzyna.tomczyk@wat.edu.pl (K.T.)

**Keywords:** brass, copper, zinc, passivation, oxidation, anodizing, Pourbaix diagram, carbon dioxide, hydrogen

## Abstract

Brasses are well-known structural materials, and their electrochemistry seems to be known. However, the formation of nanostructured anodic oxides on brasses is still not common and researched enough. Despite the electrochemical oxidation or anodization of copper and zinc being well-recognized and known in the scientific community, there is a lack of a satisfactory amount of research on brass anodizing. Both copper and zinc can passivate in neutral and alkaline electrolytes, and also the mechanism of the nanostructured oxide growth of both seems to be similar. In this review, much effort was put in to gather the information on the protocols on the electrochemical oxidations of brasses and their applications. Usually, the effects of electrochemical oxidation allow us to obtain nanostructured surfaces made of mixed Cu and Zn species. The formation of such composite nanostructures allows us to apply them in such emerging applications as photocatalytic organic pollutant decomposition, photoelectrochemical hydrogen generation, electrochemical carbon dioxide reduction reactions, or electrochemical methanol oxidation.

## 1. Introduction

The electrochemical oxidation of metals and alloys allows the formation of nanostructured semiconductors. However, the origin of the electrochemical oxidation of metals with the formation of an adherent oxide layer lies in the corrosion protection of aluminum alloys [1,2]. Since tools such as scanning electron microscopy have been developed, it has been noticed that the two-electrode oxidation and anodizing of aluminum and its alloys provide a nanoporous oxide with the pores parallel to each other and perpendicular to the oxidized surface of the substrate material [3,4,5]. In 1995, Masuda and Fukuda delivered a breakthrough when they reported the formation of a hexagonally arranged, honeycomb-like array of the nanoporous anodic alumina, which triggered an incredible amount of research worldwide [6]. Since then, much fundamental research on anodic alumina has been reported on such subjects as the impact of the anodizing conditions on the morphology of the formed oxides [7,8,9,10,11,12,13,14,15,16,17,18,19], the arrangement of the pores [20,21,22,23,24,25,26], the formation of pores with unique shapes [27,28,29,30], or on branched pores [31,32,33]. Since then, the application of the hexagonally arranged alumina as a template for nanofabrication has resulted in the formation of nanowires [34,35,36,37,38,39,40,41,42,43], nanotubes [38,44,45], nanofibers [46], and nanodots [47,48] made of various materials like metals [34,35,36,37,38,44,45], oxides [39], salts [40,41,48], or polymers [42,43,46], which have contributed to numerous ways in such applications as electrochemical sensing [43,46], water electrolysis [45], catalysis [29], and semiconductive devices [48].

Simultaneously, metals other than aluminum have been anodized, including Fe [49], Zn [49,50,51], Zr [49,52], W [53,54], Sn [55,56,57], Ti [58,59,60], Ta [61], and Nb [62], which have contributed to such emerging applications as wastewater treatment [49,62], microplastic removal [59], and photocatalysis [54,58], including “green hydrogen” generation [53,58].

Furthermore, alloys like stainless steel [63,64,65] or iron aluminides [66,67,68,69] have been anodized, and it has been shown that also in these cases nanostructured anodic oxides are formed.

Most of the electrochemically grown oxides generally have a couple of common features: the oxides are nanoporous or nanotubular (except anodic ZnO—made of nanowires [49,50,51]), amorphous when as-obtained (except ZrO_2,_ which sometimes might be crystalline [52]), and have fixed stoichiometry.

**Figure 1:**
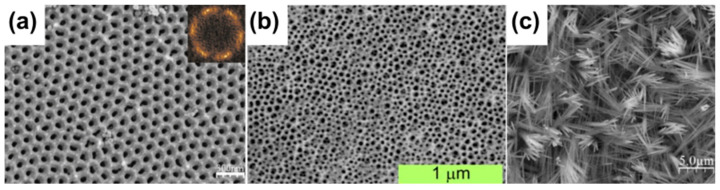
FE-SEM images of nanostructures formed by the electrochemical oxidation of aluminum (**a**), zirconium (**b**), and copper (**c**). Reprinted with permission from Elsevier [7,52,74].

Electrochemical oxidation, or anodizing, of copper provides quite unique features when compared to other oxides [69]. The most remarkable is the chemical composition of the products of the electrochemical oxidation of copper—after the electrochemical oxidation/anodizing, copper (I) oxide, copper (II) oxide, and copper (II) hydroxide are present on the surface, due to the much more complex mechanism of the oxide growth when compared to simple field-assisted etching [70,71]. In contrast, other anodic oxides are classical daltonides with maintained stoichiometry, like Al_2_O_3_ (Figure 1a), TiO_2_, or ZrO_2_ (Figure 1b). Furthermore, the majority of the anodic oxides are amorphous when as-synthesized oxides are investigated with XRD. Depending on the synthesis method, the copper electrochemical oxidation results in the formation of cubic cuprite and monoclinic tenorite [70,71]; sometimes also crystalline Cu(OH)_2_ is detected. But the most interesting fact is that even incorporated species, like carbonates, form crystalline phases such as malachite [72,73]. But the most remarkable feature of the electrochemically grown oxides on copper is morphology. The majority of the anodic oxides have nanoporous or nanotubular morphology, while the electrochemical oxidation of copper allows the formation of nanorods or nanoneedles [70,71,72,73,74] (Figure 1c).

Similarly, the electrochemical oxidation of zinc provides different types of nanostructures. The formed zinc oxides are made of nanowires; however, the anodically formed zinc oxides are amorphous, and only heat treatment allows the formation of crystalline phases [75,76,77]. Additionally, anodically grown ZnO is a typical daltonide with fixed stoichiometry.

**Figure 2:**
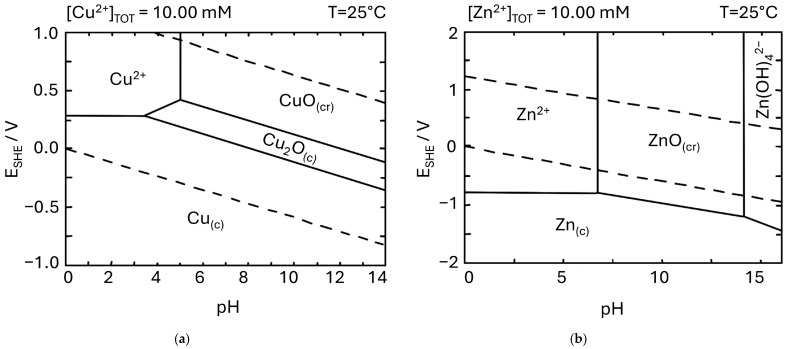
Pourbaix diagram for copper (**a**) and zinc (**b**).

Both metals, copper and zinc, have similar pH ranges in the Pourbaix diagrams (Figure 2), indicating passivity. The optimum pH for passive oxide formation is within the range of 6–14 for both elements. Therefore, it triggers questions about brass electrochemical oxidation. In terms of corrosion, brass is a typical example of selective corrosion, where zinc corrodes due to the lower electrochemical potential, while copper remains immune (dezincification). In the case of properly adjusted electrolytes for passive film growth, electrochemical formation of the nanostructures was shown to be possible.

The major motivation of this paper is to review the studies where nanostructures were formed via brass electrochemical oxidation. The nanostructured, mixed oxides are demanded in such emerging applications as photocatalysis and electrochemical catalysis. Therefore, such a simple way of performing surface nanostructurization is promising and requires much more attention from the scientific community.

## 2. Electrochemical Oxidation of Copper

### 2.1. Strategies for Copper Electrochemical Oxidation

As mentioned above, copper passivates with the formation of nanostructures in electrolytes with an alkaline pH (Figure 2a). The first successful attempts were reported over a decade ago. Allam and Grimes [78] were among the first to report the formation of various morphologies via copper anodizing. The authors reported that, they useda two-electrode cell and a simple anodizing setup in their research. Depending on the pH of the electrolyte or applied additives, such as fluorides or chlorides, variety of nanostructures, including nanowires, nano leaves, nanopillars, or dendrites were formed. Oyarzún Jerez et al. demonstrated the formation of porous oxide by copper anodizing in fluoride-rich media, similar to those in which titanium is anodized [79]. However, the most interesting aspect from the application point of view is the formation of high-aspect-ratio nanoneedles [69].

**Figure 3:**
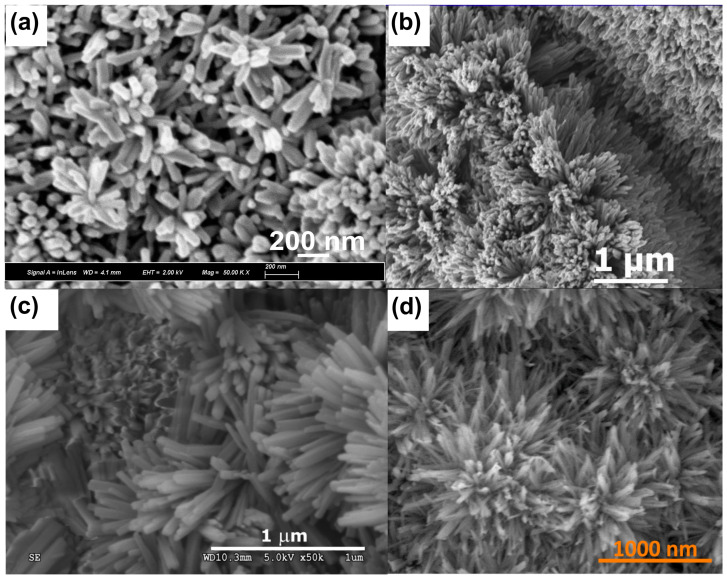
FE-SEM images of nanostructures grown by copper electrochemical oxidation in 0.01 M KHCO_3_ (two-electrode system, 50 V) [70] (**a**); 0.01 M NaHCO_3_ (two-electrode system, 25 V) [80] (**b**); n 0.1 M Na_2_CO_3_ (two-electrode system, 31 V) [81] (**c**); 0.1 M NH_4_HCO_3_ (potentiostat, 400 mV vs. Hg|HgO) [72] (**d**). Reprinted with permission from Elsevier and copied from MDPI from articles under CC BY 4.0.

In general, there are currently two major approaches in copper electrochemical oxidation: a typical, two-electrode anodization and electrochemical oxidation using a potentiostat for more detailed, mechanistic investigations. More detailed and tabularized information on the electrochemical synthesis of the nanostructures formed on copper has been previously reported [69]; however, some recent milestones have to be mentioned (Table 1). Firstly, since the last broad review paper, numerous new electrolytes have been revealed to be efficient in copper electrochemical oxidation. Previously, NaOH or KOH was usually used, and only some additives, such as chlorides or fluorides, were applied [69,78]. Since then, the effective electrochemical oxidation of copper in such electrolytes as KHCO_3_ [70] (Figure 3a), NH_4_HCO_3_ [72] (Figure 3c), NaHCO_3_ [73,80] (Figure 3b), K_2_CO_3_ [71], or Na_2_CO_3_ [81] has been performed based on the Cu Pourbaix diagrams (Figure 2a). In all the cases, a well-developed surface area of the electrochemically grown nanostructures was achieved. In both cases, either using the standard anodizing two-electrode system (Figure 3a–c) or a potentiostat allowed formation of the nanostructures (Figure 3d). However, it is apparent that in the case of the two-electrode anodization, the nanowires have a lower aspect ratio, while in the case of potentiostat longer, nanoneedle-like structures are obtained (Figure 1c and Figure 3d).

### 2.2. Applications of Nanostructures Formed by Copper Electrochemical Oxidation

In terms of the most significant applications, electrochemically grown nanostructures on copper were found to be key materials in numerous applications, including electrocatalysis (Table 1). Anantharaj et al. reported the synthesis of the nanostructured copper oxides [82,83] and their applications in the electrochemical oxidation of methanol, a key reaction in direct methanol fuel cells (DMFC). Firstly, it was found that the longer the passivation in 1.0 M KOH, the greater the methanol oxidation current density, due to the developed surface area (Table 1) [82]. Next, the same authors continued their investigations and subjected copper to passivation in electrolytes with various pH levels, ranging from 12.0 (0.01 M KOH) to 14.8 (6 M KOH), and they revealed that the greater the pH of the electrolyte, the greater the copper (II) hydroxide to copper (II) oxide ratio [83]. This confirms the key role of water-soluble Cu(OH)_4_^2−^ species in forming nanostructures by redeposition, as postulated in [70]. Moreover, it was found that the greater the Cu(OH)_2_ to CuO ratio, the lower the charge transfer resistance (estimated by electrochemical impedance spectroscopy, EIS) and, consequently, the higher the methanol oxidation current density. Therefore, electrochemically formed nanostructures on copper were found to be straightforward for synthesizing materials for DMFC.

Electrochemically grown copper oxide nanostructures also provide significant contributions to such emerging issues like global warming. It was found that electrochemically grown CuOx nanostructures are great catalysts for electrochemical carbon dioxide reduction into hydrocarbons and alcohols. For example, Lee et al. report that the nanostructures formed in 3 M KOH by the application of constant current density (10 mA/cm^2^; Table 1) were efficient and stable catalysts for CO_2_ reduction into ethylene [84]. Most importantly, plain copper foil’s selectivity towards ethylene was dropping in time, from ca. 17% to ca. 6% after 80 min of the reaction, while the anodically grown nanostructures allowed the authors to obtain a selectivity of 30% over time, which suggests the great stability of the nanostructures as catalysts. What is even more important, in contrast to the plain foil, reactions run on the anodically grown nanostructures resulted in almost no methane (1.3% Faradaic Efficiency/selectivity), which has proven that the oxide-derived nanostructures facilitate the formation of the C-C bonding that is crucial to this application. The Faradaic Efficiency/selectivity data are also supported by the chronoamperometric curves, which also indicate that the current registered during CO_2_ reduction on anodized copper is stable, in contrast to the current density registered on the plain Cu foil. These data are in agreement with results obtained by Giziński et al. for copper anodized in 1.0–3.0 M NaOH [85]. It was also found that the greater the concentration of NaOH as the synthesis electrolyte, the greater the electrochemically active surface area (ECSA) of the surface, and the greater the values of the current densities registered during the CO_2_ reduction reaction. What is even more important is that despite the grown nanostructures being made of copper oxides and copper (II) hydroxide, after the reduction in carbon dioxide, the developed morphology is inherited, which results in a stable current density during the catalysis. Xie et al. reported the formation of nanoflowers obtained by pulse Cu passivation in a more ambient electrolyte, composed of 200 g/L of NaCl and 4 g/L of NaOH (Table 1) [86]. It was found that also on these nanostructures the electrochemical reduction of carbon dioxide leads to the formation of methane and ethylene. Importantly, the selectivity (Faradaic Efficiency) of two competing reactions, hydrogen generation and the carbon dioxide reduction reaction, indicates that the electrochemically grown nanostructures have a much greater affinity for reducing carbon dioxide. Namely, after 2 h of CO_2_ reduction reaction, FE for H_2_ formation rises to 100%, while FE towards methane, ethene, and formic acid formation drops to zero for plain copper. Simultaneously, for the electrochemically grown nanostructures, even after 9 h of reaction, H_2_ FE is below 40%, while the FEs of the formation of formic acid, methane, and ethene are at the levels of 70, 2.5%, and 2.5%, respectively. Recently, a significant breakthrough in CO_2_ reduction was reported by Sun et al., where electrochemically grown nanostructures were grown not on a plain Cu foil but on a mesh in a gas-diffusion electrode [87]. What is important to mention is that the only way to scale up the carbon dioxide reduction reaction to industrial needs is via the application of gas-diffusion systems. Also, the formed nanoneedles were chemically covered with 1-octadecanethiol to improve the hydrophobicity of the surface. The most remarkable is the Faradaic Efficiency of the C_2+_ products (products that have at least one C-C bond, i.e., ethene, ethanol, n-propanol, etc.) of the CO_2_ reduction reaction, which was as high as 87%. Therefore, such milestone results promise significant progress in the most crucial issues to be solved by the researchers.

The electrochemical oxidation of copper also contributed to the formation of catalysts for the photoelectrochemical hydrogen generation via water splitting. One of the problems is the stability of the catalysts, which is sometimes poor due to the photocorrosion. Zhang et al. [88] reported that coating the electrochemically grown Cu_2_O, or Cu(OH)_2_ can be completed by immersion in glucose and subsequent carbonization in the ambient atmosphere. This treatment allows the maintenance of stability due to the carbon, which acted as a photocorrosion inhibitor with a simultaneous water-splitting efficiency. Oyarzún et al. applied fluoride and ethylene glycol-based electrolytes, typical for d-electronic metal anodizing [79,89]. They have tested various electrolytes with various contents of water, namely 10, 5, and 1% (Table 1). It was shown that the greater the water content, the longer the nanoneedles formed. What is more, when water content was the smallest, the needles were made of Cu_2_O (100 nm or shorter), while the water content was greater; also, CuO was present in the nanostructures. It was found that the obtained nanoneedles are efficient photocatalysts for organic compound degradation. Methylene blue was used as a model organic compound, and it was found that for the Cu_2_O/CuO nanoneedles photodecomposition efficiency is as high as 88% and was reported to be maintained as high as 87% even after five cycles. For the nanoneedles formed in electrolyte containing 1% H_2_O, only Cu_2_O was formed, and the photodegradation efficiency was only 74%.

Therefore, copper electrochemical oxidation allows the formation of high-aspect-ratio nanostructures with a great impact on emerging applications, including water splitting and carbon dioxide reduction into organic compounds.

## 3. Electrochemical Oxidation of Zinc

### 3.1. Strategies for Zinc Electrochemical Oxidation

**Figure 4:**
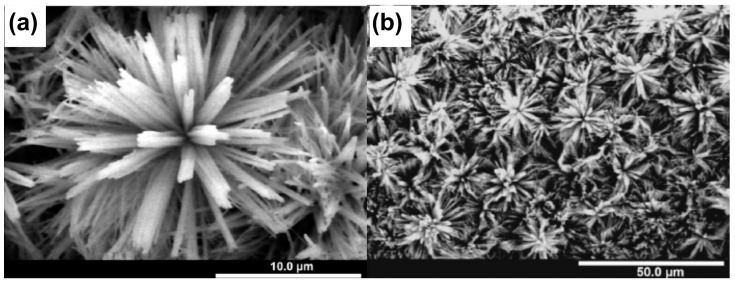
FE-SEM images of nanostructures grown by zinc anodizing 50 mM NaHCO_3_ at 10 V (**a**) [76], and 10 mM NaHCO_3_ at 10 V (**b**) [95]. Reprinted with permission from Elsevier.

As can be seen in the Pourbaix diagram, zinc has a similar passivation range as copper, ranging from ca. 7 to ca. 13 (Figure 2b). Thus, the most recommended electrolytes are also bases, like NaOH or KOH, as well as salts with alkaline hydrolysis, like the carbonates or bicarbonates of I group metals. One of the pioneering studies in the field of zinc anodizing was reported by Kim and Choi [90]. They reported the anodization of zinc in sulfuric acid at various concentrations in pure ethanol. Of course, ethanol is less polar than water; therefore, sulfuric acid dissociation is not as effective as in water, and, as a result, the ethanoic solution of sulfuric acid has a higher pH. The authors have investigated 0.2, 1.0, 2.0, and 4.0 M H_2_SO_4_ in ethanol and found that only at specific concentrations and operating conditions are ZnO stripes formed. Very often, corrosion is a competing process to passivation, which is in agreement with the Pourbaix diagram (Figure 2b). Same year, Ono et al. published results on Zn anodization in acidic and alkaline electrolytes [91]. It was found that much better results were obtained when 0.2 M NaOH (thick, porous oxide) was used as an electrolyte than when 0.3 M oxalic acid was used. In both cases, anodizing resulted in semi-crystalline oxide—the as-obtained samples were used to give some minor reflections when XRD was performed; however, annealing of the samples at 300 °C for 1 h allowed the formation of a fully crystalline material, confirmed by numerous reflections in the XRD patterns. Mah et al. reported Zn anodizing in NaHCO_3_-based electrolytes at various operating conditions [75]. This comprehensive study shows a couple of important conclusions: (i) nanostructured oxides tend to grow in pits, (ii) the concentration of the electrolyte and anodization time allow structures to evolve from dandelion-like nanostructures to a network of nanowires, (iii) the mechanism of growth is complex and involves the formation of intermediate compounds containing electrolyte anions, such as Zn_5_(OH)_6_(CO_3_)_2_, and (iv) annealing allows the formation of crystalline ZnO without the intermediate stages. Similar conclusions were drawn by Zaraska et al. [92] (Table 2). They anodized Zn in 5 mM NaHCO_3_, at 10 V and obtained semiconductive (band gap 3.20–3.23 eV) nanowires made by wurtzite after annealing. The most important finding is that the high aspect ratio is made of nanocrystals with a diameter of up to 12.5 nm—the greater the annealing temperature, the greater the crystallites. However, independently from the crystallite size, the band gap of the nanowires is in the above-mentioned range. In a further paper, Zaraska et al. reported a systemic study of the influence of operating conditions, including the concentrations of applied electrolytes, the voltage, and anodization time on the growth of the ZnO nanowires (Table 2) [76]. A very important mechanistic insight—a sequence of formation of the ZnO precursors, much more detailed than in the previous case—was reported [75]. The electrochemical oxidation of zinc ejects Zn^2+^ cations into the electrolyte that turn into coordination ions, such as [Zn(H_2_O)_6_]^2+^ and, in mildly alkaline electrolytes, such as aqueous solutions of carbonates, and which can turn into [Zn(H_2_O)_5_(OH)]^+^ or [Zn(H_2_O)_4_(OH)_2_]. In the presence of carbonate anions, water molecules can be substituted by carbonate anions as ligands, and species such as [Zn_x+y_(OH)_2x_(CO_3_)_y_] are formed. These serve as the precursors of ZnO, especially when, after anodizing, annealing is conducted. Zamora-Peredo et al. reported similar results—they anodized Zn in 50 mM NaHCO_3_ at various voltages for various times and also annealed the obtained nanowires at various temperatures (Table 2) (Figure 4a). They found a significant increase in the thickness of the nanowires with the time of anodization, which is quite important for tuning their morphology.

**Table 2:** Information on the exemplary Zn anodization synthesis protocols.

Passivation System and Procedure	Remarks	Reference
Various concentrations of H_2_SO_4_ in ethanol, 1–40 V	Sulfuric acid concentration in ethanol ranged from 0.2 to 4.0 M; various morphologies like stripes were obtained; corrosion was competing with anodizing.	[90]
0.3 M (COOH)_2_, or 0.2 M NaOH, 100 A/m^2^, 20 °C, 2 min	Both acidic and alkaline electrolytes were applied. Nanostructured oxide was formed when NaOH was applied as an electrolyte.	[91]
1–100 mM NaHCO_3_ and ethanol in 10:1 *v*/*v* ratio, 1–10 V, 1 s–30 min, RT	The nanowires were made of amorphous precursors, such as Zn_5_(OH)_6_(CO_3_)_2_. Annealing allowed the formation of crystalline ZnO. Carbonates were involved in the formation of the nanowires.	[75]
5 mM NaHCO_3_, 10 V, RT	After anodizing, samples were annealed at temperatures ranging from 100 to 300 °C for 2 h; high-aspect-ratio nanowires were obtained as long as 20 µm with diameters ranging from 100 to 200 nm. The band gap of the obtained wurtzite nanowires ranged from 3.20 to 3.23 eV.	[92]
5–50 mM NaHCO_3_ or NH_4_HCO_3_, 5–20 V, 1–30 min	Samples after anodizing were annealed in the air, 200 °C, 2 h; the longer the anodization time, the more densely packed the nanowires.	[76]
50 mM NaHCO_3_, 10 V (1–5 min), 2 min (2.5–15 V)	Annealing at 100–400 °C was performed; Raman spectra were recorded.	[93]
50–230 mM KHCO_3_, 5–15 V, 5–20 min	Annealing at 350 °C for 1 h was performed; UV–visible diffuse reflectance spectra (DRS) were recorded.	[50]
0,1 M KOH, 3–15 V, 25 °C, 30 min	Oxide with a highly developed surface area was obtained, but not made of nanowires.	[94]
NaHCO_3_-KHCO_3_, 20 V, RT, 20 min	Annealing at 400 °C for 70 min was performed; various concentrations of sodium and potassium bicarbonates were applied.	[51]
10 mM NaHCO_3_, or 10 mM Na_2_CO_3_, or 5 mM NaHCO_3_ + 5 mM Na_2_CO_3_, 10 V, 10 min	ZnCO_3_⋅H_2_O or Zn(HCO_3_)(OH) is formed on the Zn surface during anodizing.	[95]
50 mM KHCO_3_, 2, 40, or 60 V, 30, 60, or 120 min	Samples after anodizing were annealed in the air, 300 °C, 1 h; efficient photocatalyst for methylene blue decomposition, up to 87.3%	[96]
1 M NaOH, 2 V, 2–120 min, RT	Some of the samples were annealed in the air at 200 °C; the mechanism of growth involves the formation of Zn(OH)_4_^2−^ as an intermediate; the Incident Photon to Current Efficiency exceeded 16% for the optimized samples.	[97]
0.5 mM NaHCO_3_, 5 V vs. Hg|HgO, 2, 30, or 40 min	Three-electrode system with a potentiostat was applied; Zn/ZnO served as the anode in high-capacity batteries.	[98]

### 3.2. Applications of Electrochemically Grown ZnO

Mateen Tantray reported Zn anodization in KHCO_3_ [50] (Table 2). It was also found that the as-anodized samples obtained in this electrolyte have some crystallinity; however, annealing improves it. The most important part is that the authors recorded the photocurrent response of the samples, and for the optimized conditions, the greatest photocurrents recorded were as high as 30 μm/cm^2^. This shows that the anodically formed ZnO nanowires might be promising materials for photocatalytic applications. Masuda et al. used a much more alkaline electrolyte for zinc anodization—0.1 M KOH [94] and the obtained oxide was nanostructured but did not consist of the nanowires (Table 2). The mechanism of growth of the oxide could be expected to be different due to the possible Zn(OH)_4_^2−^ corrosion according to the Pourbaix diagram (Figure 2b). The corrosion phenomenon was also responsible for the obtained rough metal/anodic oxide interface. Mateen Tantray reported Zn anodization in mixed electrolytes composed of KHCO_3_ and NaHCO_3_. It was found that the greater the concentration of salts in the electrolyte (up to 25 mM) the greater the close packaging of the formed nanostructures [51] (Table 2). Mika et al. reported Zn anodization in 10 mM NaHCO_3_, 10 mM Na_2_CO_3_, or a mixture of 5 mM NaHCO_3_ + 5 mM Na_2_CO_3_, (10 V, 10 min) [95] (Table 2). What is important is that the authors reported the speciation diagram for Zn species, and regarding the rich amount of characterization techniques, including XPS and Raman spectroscopy, they have concluded that there is one more intermediate phase in forming the nanostructures (Figure 4), namely, ZnCO_3_⋅H_2_O or Zn(HCO_3_)(OH) [95], confirming the key role of the carbonates in the formation of the nanowires. Oksuz et al. also reported Zn anodization in a carbonate electrolyte; however, they applied much greater anodizing voltages and a much longer duration of the processes (Table 2) [96]. Typically, Zn is anodized at low voltages, usually up to 10 V, while the authors anodized it at 20, 40, or 60 V (Table 2). Nevertheless, also in the case of higher voltages, the oxide was formed, and, surprisingly, the best, closely-packed nanowires were obtained at 40 V. Oksuz et al. also used the obtained nanostructures as catalysts for the photodegradation of methylene blue. It was found that the samples providing the best photocatalytic efficiency (over 87%) were synthesized at 20 V for 30 min. Mika et al. reported the fabrication of the “*lawn*”-like ZnO nanostructures via zinc foil anodization in 1 M NaOH [97] (Table 2). In the carbonate-free electrolyte, a different mechanism for the growth of the oxide nanostructures is considered—it was found that water-soluble intermediate Zn(OH)_4_^2−^ plays a crucial role in the ZnO precipitation on the surface. It was also noticed that the as-obtained and annealed samples have comparable photoelectrochemical properties. Incident Photon to Current Efficiency (IPCE) was at the level of ca. 16–17% for both types of samples. It is important to mention that the best IPCE was for samples anodized for 30–60 min.

Hui et al. reported the passivation of zinc in a three-electrode system [98] (Table 2). Also, in this case, densely packed ZnO nanowires were obtained. The obtained Zn/ZnO electrodes were coupled with N, P-co-doped hard carbon (NPHC) cathodes and served as batteries. It was found that the performance is competitive compared to the most recent approaches. The high specific capacity of the cell, as high as 110.3 mAh g^−1^ at 0.1 A g^−1^, was accompanied with a ca. 100% Coulombic efficiency after 20,000 cycles.

Zinc anodization allows for the formation of nanostructured oxides in a simple way, which might have numerous applications in photoelectrochemistry and energy storage devices.

## 4. Electrochemical Oxidation of Brass—Synthesis and Applications

### 4.1. Brass as a Substrate for Electrochemical Oxidation

**Figure 5:**
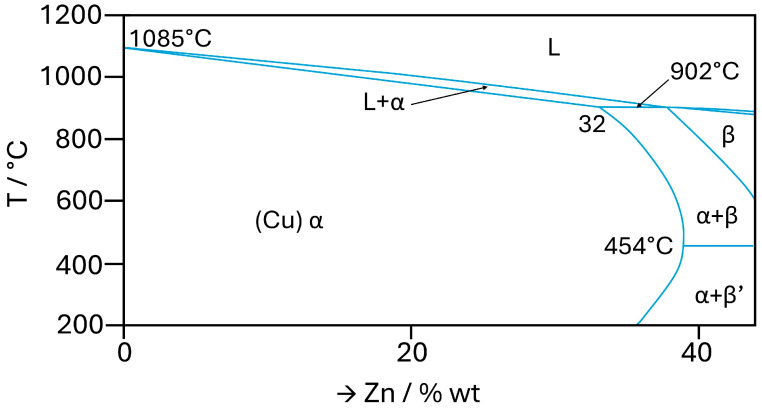
Cu-Zn binary phase diagram for α-brass range.

Copper and zinc are neighbors in the periodic table of the elements and thus have similar atomic radii; however, these metals crystallize in different systems. Copper is face-centered cubic (FCC), while zinc is hexagonally close-packed (HCP). Thus, both these metals do not obey Hume-Rothery’s rules; however, they are soluble to a certain extent (Figure 5). From the anodization or general electrochemical oxidation point of view, the most interesting cases are the one-phase brasses, made of a solid solution of zinc in copper. The chemical homogeneity of the alloy allows the formation of nanostructured oxides. In practice, researchers anodize brasses with a zinc content of up to 37%. There are two major approaches to electrochemically oxidized brass: a traditional, scalable, two-step approach known as anodizing and passivation using a potentiostat (three-electrode approach); however, both provide similar effects in terms of the formed oxides.

### 4.2. Electrochemical Oxidation of Brass—Strategies and Applications

Eissa et al. reported anodization of brass containing 30% Zn in 0.1 and 1.0 M NaHCO_3_ [99]. Materials with a highly developed surface area were obtained; however, nanostructures were formed only in the case of anodization in 1.0 M NaHCO_3_, followed by annealing in the air atmosphere (ca. 1.09 µm long nanowires) (Table 3). Both XRD and Raman spectroscopy confirmed the presence of both CuO, and ZnO; therefore, the formed nanostructures can combine the advantages of both oxides. These composite-oxide nanostructures were used in photoelectrochemical water splitting, and it was found that in the case of anodized brass, much greater photocurrents were acquired than in the case of pure ZnO, namely, up to 1.88 mA/cm^2^. What is more, the IPCE of the reaction conducted on this material was up to 15%. When the photoelectrochemical water splitting was running for 5 h, researchers obtained even 0.875 mmol of H_2_. Ryczek et al. anodized brass containing 32% of Zn (Table 3). In this case, relatively high values of the anodizing voltages were applied, ranging from 30 to 60 V (0.1 M NaOH) [100]. Also, in this case both CuO and ZnO were detected (XRD, Raman), and a certain relation was observed: the greater the anodizing voltage, the greater the Cu to Zn ratio, ranging from 2.6 (30 V) to 3.6 (60 V), and consequently the greater the band gap, ranging from ca. 1.41 to ca. 1.63 eV. The mixed anodic oxide was made of nanoparticles; thus, no high-aspect-ratio nanostructures were noticed. Nami et al. reported the anodization of 35% Zn brass in 0.1 M NaOH with 0.025 M NH_4_Cl (Table 3) [101]. The oxides obtained had a developed surface area but no regularity, nor were certain specific nanostructures noticed. However, the XRD examination revealed the co-existence of a couple of crystalline phases, including Cu_2_O, CuO, ZnO, and CuCl_2_. Samples were anodized at the same voltage (12 V, Table 3), but for various times, from 15 to 45 min, and it was revealed that the longer the anodization, the greater the CuO to ZnO ratio. The developed surface area allowed the application of the obtained oxides as catalysts for organic pollutant photodegradation. As a model compound, methylene blue (MB) was used, and it was found that for the optimized samples, its degradation reached 54% after 300 min when 100 mL of 2 mg/L of MB was used. A very extensive study on the influence of the operating conditions, including the type of electrolyte and additives, as well as the applied voltage and the duration of the process, was delivered by Dezfoolian et al. [102] (Table 3). Depending on the operating conditions, various morphologies were obtained, including spheres, flake-like structures, polygons, or cubes, with morphological features ranging from hundreds of nanometers to microns.

**Figure 6:**
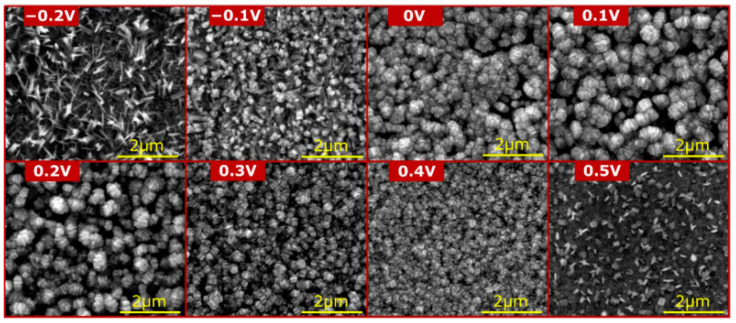
FE-SEM images of nanostructures grown by brass (Zn 37%) anodization using a potentiostat in 1.0 M NaOH at various potentials (given vs. Ag|AgCl). Copied from MDPI from an article under CC BY 4.0 [103].

**Figure 7:**
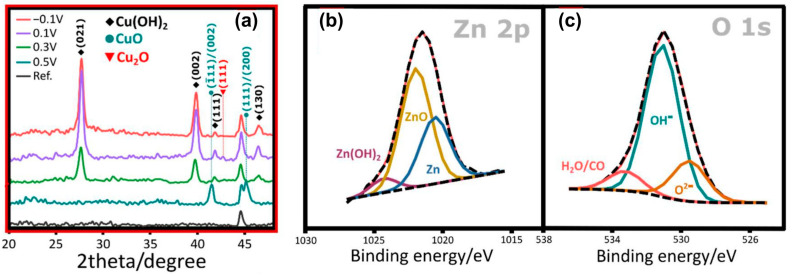
XRD patterns of brass passivated in 1.0 M NaOH at various potentials (**a**) and XPS spectra for Zn 2p (**b**) and O 1s energy range (**c**) of brass passivated in the same electrolyte at 0.1 V vs. Ag|AgCl. Copied from MDPI from an article under CC BY 4.0 [103].

**Figure 8:**
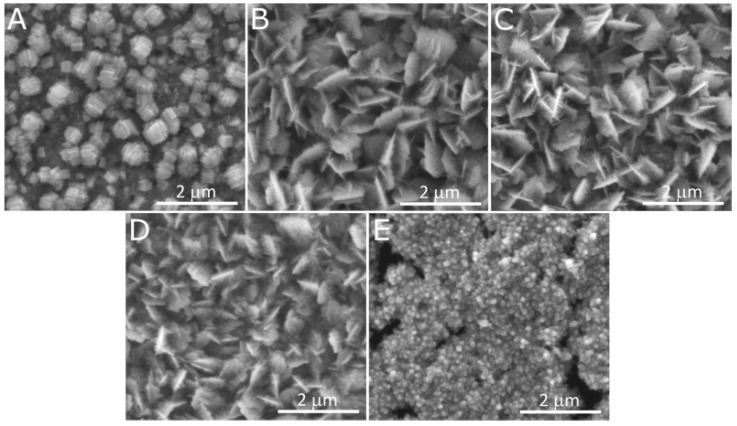
FE-SEM images of nanostructures grown by brass (Zn 37%) anodization using a potentiostat in 1.0 M NaOH containing 0 (**A**), 5 (**B**), 10 (**C**), 15 (**D**), and 30% (**E**) of glycerol at 300 mV (given vs. Hg|HgO). Reprinted with permission from Elsevier [104].

In the above-discussed papers [99,100,101,102], the authors have used a standard, two-electrode system for brass anodization (30–35.54% Zn). In all the cases, mixed oxides were obtained, and their morphology was developed, but there were no distinct nanostructures like nanorods, nanowires, nanoneedles, or nanopores. A significant change was reported by Giziński et al. [103], where 37% Zn brass was anodized in 1.0 M NaOH using a potentiostat (Table 3). It appears that the nanostructures, depending on the applied potential, are in the form of nanowires (−0.2 V and −0.1 V vs. Ag|AgCl) or nanorods agglomerated into “nanobarrels” (0–0.4 V vs. Ag|AgCl) (Figure 6). It was also found that the greater the applied potential, the smaller the nanobarrels obtained. Moreover, the samples were analyzed in terms of the chemical and phase compositions without annealing, and it was found that only copper-containing crystalline phases, mainly Cu(OH)_2_ and CuO, are present in the as-obtained samples (Figure 7a), while zinc-containing phases are present but amorphous, which was confirmed by the XPS analyses (Figure 7b,c). The obtained nanostructures were used as photocatalysts for methyl orange (MO) and provided satisfactory decomposition rates when both UV and visible light sources were applied. The composite nanomaterial allows the decomposition of MO due to the presence of ZnO, while the copper species, especially copper (II) hydroxide, hinder the recombination of the electron–hole pair. In [104], Brudzisz et al. reported the passivation of 37% Zn brass also using the three-electrode system and a potentiostat, but in 1.0 M NaOH with various amounts of glycerol added. Glycerol allowed the variation of the viscosity and conductivity of the electrolyte and was found to have a significant impact on the morphology and chemical composition of the grown passive oxides. In the electrolyte without any glycerol, typical nanobarrels were obtained (Figure 8A) [104], as in the case of previously reported research [103]. For samples passivated in electrolyte containing 5, 10, or 15 vol. % of glycerol, pellet-like structures were obtained (Figure 8B–D), while for the samples grown in electrolyte containing 30 vol. % of glycerol, nanoparticles were formed (Figure 8E). However, the most interesting are the results of the phase composition of the grown oxides. For samples passivated in electrolyte without any glycerol, mainly Cu(OH)_2_ was detected as a crystalline phase, while for the samples obtained in more viscous electrolytes, crystalline ZnO and Zn(OH)_2_ appeared (without annealing), while copper-based crystalline phases were not detected. This indicates a crucial role for glycerol in the mechanism of nanostructure formation. The most probable explanation is the formation of a water-soluble Cu/glycerol coordination compound that prevents the redeposition of Cu(OH)_2_ and the formation of a nanostructured deposit on the surface, which would be in line with the EDTA study reported previously [70]. Also, the EDS analyses revealed that the greater the electrolyte viscosity, due to the increased glycerol content, the greater the Zn to Cu ratio in the formed oxides.

What is more, the XPS analyses confirmed the presence of Cu_2_O in the formed oxides, which means that for the viscous electrolytes, only a rather amorphous cuprous oxide is formed, while the copper (II) hydroxide redeposition is blocked due to the formation of the stable coordination compound with glycerol.

**Figure 9:**
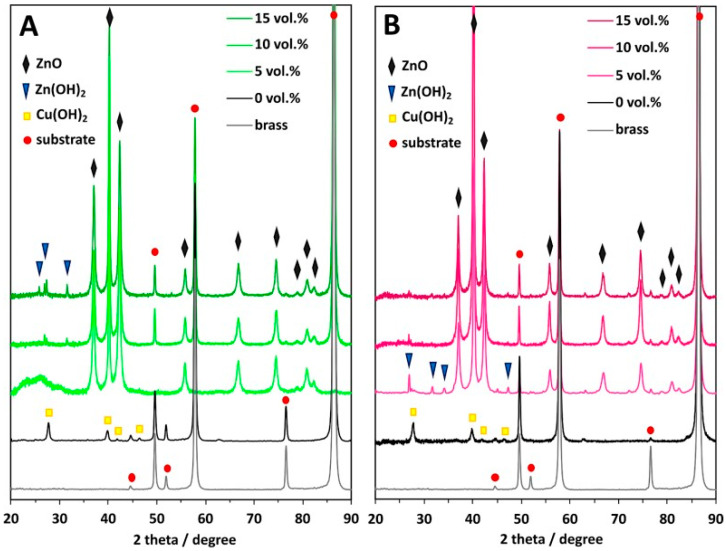
XRD patterns of brass passivated in 1.0 M NaOH 100 (**A**) and 300 mV vs. Hg|HgO (**B**) in electrolytes containing various amounts of glycerol. Reprinted with permission from Elsevier [104].

The mechanism of the growth of oxide/hydroxide nanostructures has not yet been studied. However, certain premises resulting from the data published for copper and zinc allow for drawing certain outlines. For both Cu and Zn, simple field-assisted etching does not form nanostructures, but the step of precursor redeposition is crucial in nanostructure formation. In the case of Zn, depending on the electrolyte, Zn(OH)_4_^2−^ in NaOH [97] or Zn_5_(OH)_6_(CO_3_)_2_ in carbonate solutions [75], the redeposition is crucial to form the nanostructures. In the case of Cu, hindering Cu(OH)_2_ redeposition by using EDTA demonstrated that the formation of copper (II) hydroxide on the Cu surface is crucial for nanoneedle formation on Cu [70]. Therefore, the mechanisms of the oxide/hydroxide nanostructure growth on both metals are similar. Thus, the precursor redeposition from the electrolyte is key in nanostructure formation. Hindering the redeposition of the metals allowed the morphology to evolve (Figure 8) and influenced the composition of the grown nanostructures (Figure 9). However, it is apparent that the higher the amount of glycerol, the less Cu species in the formed oxides and the greater the Zn/Cu ratio (Figure 9). It shows that the hindering of the redeposition is selective and halts mainly Cu species from redepositing on the brass surface. Nevertheless, these formed nanostructures (Figure 8) are a far cry from those formed on pure Zn (compare with Figure 4). On the other hand, it was shown that by adding glycerol, the chemical composition and morphology of the grown nanostructures can be controlled to a certain extent. Thus, the mechanism of the oxide/hydroxide nanostructures is complex and requires deepened, mechanistic study.

**Figure 10:**
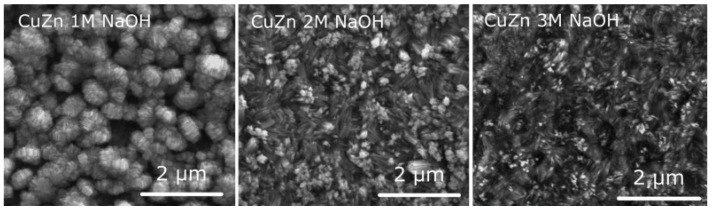
FE-SEM images of brass (37% of Zn) passivated in NaOH with various concentrations at 100 mV vs. Hg|HgO. Reprinted with permission from Elsevier [85].

Samples obtained in electrolytes with various viscosities were applied as catalysts for an electrochemical CO_2_ reduction reaction in 0.5 M KHCO_3_. It was revealed that the highest values of the recorded current densities during reactions were for samples with a barrel-like morphology (Figure 8A); that is, in the electrolyte without added glycerol. Such samples had the highest copper content and the most developed morphology. The same brass (37% wt. of Zn) was also passivated using a potentiostat, but in NaOH in various concentrations, ranging from 1.0 to 3.0 M [85]. Already the FE-SEM observations revealed the evolution of the nanostructure morphology with the electrolyte concentration, namely, an electrolyte containing 1.0 M NaOH; typical nanorods assembled in nano-barrels were obtained (Figure 10) (Table 3). However, the greater the NaOH concentration, the greater the aspect ratio of the nanorods obtained. Thus, changing the electrolyte allows for the tuning of the oxide’s morphology, which is desired in certain applications, such as in catalysis. For this reason, the electrochemically active surface area (ECSA) was measured by cyclic voltammetry in the electrochemical double-layer charging zone (where no electrochemical reaction occurs), and, surprisingly, it was revealed that the greater the NaOH concentration, i.e., the longer the nanowires, the smaller the ECSA. Moreover, during the electrochemical carbon dioxide reduction reaction experiments, this was confirmed by the greatest values of current densities recorded for brass passivated in 1.0 M NaOH. Furthermore, it was shown that after the carbon dioxide reduction reaction, the developed morphology of the brass sample is maintained; thus, the catalyst, despite the oxide being reduced (and valuable oxide-derived nanostructures being obtained), can be used again in the same application.

Due to the formation of a highly developed surface area, brass after electrochemical oxidation also finds application as a template for developing the surface area of other materials. For example, Al-Osta reported the anodization of brass in an acidic environment and obtained nanoparticle-like morphology [105] (Table 3). Thus, the nanoparticles served as a template for the further deposition of NiO. Such material was shown to have extraordinary properties as a supercapacitor, with a capacitance of up to 51.39 F/g, ca. twice greater than the standard ones. Recently, Jha et al. reported passivation of low-zinc brass (5% of Zn) using a potentiostat, with results dependent on the applied potential. In 1.0 M KOH (Table 3), they obtained previously mentioned nano-barrels (−50 mV vs. Hg|HgO) or morphology very similar to that obtained on zinc (−65 mV vs. Hg|HgO) [106]. What is interesting is that depending on the applied potential during the syntheses, XRD detects only ZnO and Cu_2_O (−50 mV vs. Hg|HgO) or ZnO, Cu_2_O, CuO, and Cu(OH)_2_ (−65 mV vs. Hg|HgO). The formed materials were utilized in electrochemical methanol oxidation, the key reaction in DMFC (direct methanol fuel cells). The authors have shown that due to the formation of the oxide nanostructures via anodization, the onset potential for methanol electrooxidation was much lower, even as low as 405 mV vs. Hg|HgO for the samples obtained at −65 mV vs. Hg|HgO. Moreover, there are many controversies about using common metals as electrodes for DMFCs due to the competitive oxygen evolution reaction (OER). The authors also performed experiments in the reference electrolyte (1.0 M KOH without methanol), but without methanol, and have empirically proven that on these materials methanol oxidized at much lower overpotentials than oxygen; therefore, the anodized brass might be considered as a valuable material for DMFCs.

The amount of varying approaches in brass electrochemical oxidation allows us to draw compact, summed-up conclusions on the synthesis (Table 4). Namely, the application of both the potentiostat and three-electrode set-up, or a simple DC-power supply in the two-electrode system, allows the oxidation of brass, resulting in the formation of the oxides. However, when a potentiostat is applied, much more uniform nanostructures are obtained. Furthermore, according to the Pourbaix diagram for Cu and Zn, the most suitable pH levels for the oxide formation electrolyte are alkaline, from mildly alkaline, such as the aqueous solutions of carbonates, to strongly alkaline, such as 3 M NaOH. Also, there are individual reports on the additives, and it was found that glycerol has a strong impact on the chemical composition and morphology of the grown oxide.

The reviewed publications focus on fundamental research; however, they provide materials that could significantly contribute to current science and technology. When scaling up, researchers will encounter numerous challenges that sooner or later will have to be faced. For example, the morphology of high-aspect-ratio nanoneedles provides a developed surface area; however, the high-aspect-ratio nanostructures cause inflammations when inhaled, similar to asbestos. Furthermore, during the syntheses, d-electronic metals, copper, and zinc are ejected into the electrolyte; thus, the waste treatment will also be an important part of the problem when a large-scale anodizing/passivation plant is run. Furthermore, the long-time stability and storage of the formed nanomaterials will also have to be investigated. Finally, in aluminum alloys anodizing, energy consumption is a crucial factor that makes the business profitable or not [107]. Also, in the case of brass passivation/anodizing, energy consumption will be taken into account. The current state-of-the-art shows that much more uniform nanostructures are formed using low potentials; nevertheless, two-electrode anodizing is much more scalable.

## 5. Summary and Further Challenges

The electrochemical oxidation of copper and zinc is quite a common method of oxide nanostructure formation. In both cases, high-aspect-ratio nanoneedles or nanowires might be obtained. For both metals, the Pourbaix diagrams show passivation in alkaline and neutral solutions. Furthermore, for both metals, the electrochemical formation of the oxide nanostructures is complex and involves the formation of various intermediates. Those similarities encouraged scientists to put some effort into brass passivation. Depending on the applied electrochemical oxidation method, various mixed-oxide nanostructures, including nanorods and nanowires, are formed. Quite often, after electrochemical oxidation, some copper species are crystalline, while zinc species sometimes tend to be amorphous. However, such composite nanostructures, despite a still-low number of published results, found applications as catalysts for organic pollutant photodegradation, active electrodes for photoelectrochemical water splitting, electrochemical carbon dioxide reduction reactions or electrodes for electrochemical methanol oxidation. Therefore, electrochemical oxidation, or the anodization of brasses, is still an uncovered method of nanostructured composite formation, promising various achievements in the emerging applications. There are also a couple of missing pieces of the puzzle, including the mechanistic understanding of the formation of mixed oxides by electrochemical oxidation, or knowledge about the evolution of the grown oxides’ morphology and composition with the chemical composition of the alloy. For this reason, there is a need for a fundamental understanding of the growth mechanism of the oxide nanostructures of the brasses, including the balance between formed water-soluble ions and the precursors and the redeposited species. This would allow an understanding of how to steer the chemical composition, crystallinity, and morphology of the grown oxides. Tailoring those features in such composite nanostructured materials will probably contribute significantly to all the mentioned fields, including photocatalysis, electrochemical carbon dioxide reduction reaction, or electrochemical methanol oxidation for fuel cells.

Electrochemical oxidation of brasses is an elegant and scalable method of nanostructure formation. Currently, there are some applications that could be scaled up, especially in terms of the photocatalytic decomposition of the organic water pollutants or the formation of electrodes for direct methanol fuel cells, where more complex problems related to such phenomena, such as mass transport in electrochemical carbon dioxide reduction reactions, do not have to be tackled.

## Figures and Tables

**Table 1:** Information on the most recent applications of copper nanostructures formed via electrochemical oxidation.

Passivation System and Procedure	Remarks/Applications	Reference
Potentiostat; 1.0 M KOH, −0.06 V vs. Hg|HgO, 10–100 s	At 0.60 V (methanol oxidation) vs. Hg|HgO, after passivation, current density increased over 10 times—methanol oxidation	[82]
Potentiostat; 0.01–6.0 M KOH, 0.864 V vs. RHE	It was found that the greater the pH the faster the nanostructures grow, and the greater the Cu(OH)_2_ to CuO ratio; for the samples obtained in the highest pH, the highest value of methanol oxidation current density oxidation was registered—methanol oxidation	[83]
NA; 3 M KOH, 10 mA/cm^2^, 60 s	It was found that selectivity towards ethylene formation is much greater than for the plain foil, accompanied by great stability—electrochemical CO_2_ reduction into ethylene	[84]
Potentiostat; 1.0–3.0 M NaOH, 0 or 100 mV vs. Hg|HgO, RT, 20 min	Cu was electrochemically oxidized at various operating conditions. It was found that the greater the electrolyte concentration, the greater the ECSA (electrochemical surface area) and the higher the current densities recorded during the carbon dioxide reduction reaction—electrochemical CO_2_ reduction	[85]
Potentiostat; 200 g/L of NaCl + 4 g/L of NaOH; pulse passivation (0.2 mA/cm^2^ for 10 s followed by 0 mA/cm^2^ for 2 s), 2160 s	Performance of electrochemically grown nanostructures vs. plain copper plate in carbon dioxide reduction into formic acid, methane, and ethene was compared: it was shown that selectivity toward carbon on low-oxidation-state products is much better for the electrochemically grown nanostructures, even after 9 h of reaction—electrochemical CO_2_ reduction	[86]
NA; 2 M KOH, 4 mA/cm^2^, 0 °C, 30 min	Electrochemically grown copper oxides served in Gas Diffusion Electrodes (GDE) as an active material for CO_2_ reduction; scalable technology has been reported; selectivity to C_2+_ products was as high as 87%—electrochemical CO_2_ reduction	[87]
Two-electrode; 3 M NaOH, 10 mA/cm^2^ (cupric hydroxide), or 0.2–5.0 mA/cm^2^ (cuprous oxide) 25 °C, 30 min	Photocorrosion performance of catalysts for H_2_ generation: CuOx nanostructures were coated with carbonized glucose, which improved stability—hydrogen generation (water splitting)	[88]
Two-electrode; 10 V, 5 °C, 3 min	Various electrolytes were applied: 0.1% NH_4_F in ethylene glycol with 10%, 5% H_2_O, or 1% H_2_O with 0.1 M KOH or 0.1 M NaOH. The greater the water content in the electrolyte, the longer the nanoneedles. Methylene blue (MB) was subjected to photodegradation on the formed materials; the longer nanoneedles composed of both Cu_2_O and CuO were found to provide the highest degradation efficiency, equal to 88%	[89]

**Table 3:** Experimental protocols of various brasses’ electrochemical oxidation.

% Zn	Passivation System and Procedure	Remarks	Reference
30%	Two-electrode cell, 0.1 M or 1.0 M NaHCO_3_, 12 V, RT, 5 min	Samples were annealed in an air atmosphere at 450 °C for 3.5 h; applied in photoelectrochemical water splitting; IPCE at ca. 400 nm was ca. 15%	[99]
32 ± 1% (EDS)	Two-electrode cell, 0.1 M NaOH, 30–60 V	Obtained samples were annealed at 200 °C for 2 h; nanocrystalline mixed oxide was formed	[100]
35%	Two-electrode cell, 0.1 M NaOH + 0.025 M NH_4_Cl, 12 V, 25 °C, 15, 30, or 45 min	A developed surface area, nanocrystalline CuO-ZnO composite was formed, which was found to be a successful catalyst for methylene blue photodegradation	[101]
35.54% (EDS)	Two-electrode cell, 0.05–0.3 M NaOH or KOH, 3–24 V, 15–120 min	The nanostructures were obtained in 0.1 M NaOH at 12 V for 60 min and 0.2 M KOH at 6 V for 60 min. Both CuO and ZnO were detected by XRD	[102]
37%	Three-electrode system, 1.0 M NaOH, from −200 to 500 mV vs. Ag|AgCl, RT	The impact of the passivation potential on the morphology of the formed passive oxides was investigated; photodegradation of methyl orange was performed	[103]
37%	Three-electrode system, 1.0 M NaOH, from 100 to 300 mV vs. Hg|HgO|3 M KOH, RT; from 0 to 30 vol. % of glycerol was added into electrolytes	In the study, the impact of the glycerol on the morphology and composition of the grown nanostructured passive films was investigated; the grown nanostructures were used as a cathode in the electrochemical carbon dioxide reduction reaction, and it was revealed that the best samples are the ones obtained in 1.0 M NaOH without additives	[104]
37%	Three-electrode system, 1.0–3.0 M NaOH, 0 or 100 mV vs. Hg|HgO|3 M KOH, RT	Cu, CuAg_10_, and Cu_63_Zn_37_ were oxidized using a potentiostat; the samples were examined before and after the electrochemical carbon dioxide reduction reaction to compare systematically the chemical composition and morphology before and after the reaction	[85]
NA; “Cu-Zn”	Two-electrode system, 1.0 M (COOH)_2_, 40 V, RT, 25 min	Cu-Zn anodization was used to increase the surface area of deposited NiO; developed morphology	[105]
5% Zn (ASME SB36 C210)	Three-electrode system, 1.0 M KOH, −0.05, and −0.065 V vs. Hg|HgO, RT, 125 s	Depending on the applied potential, different morphologies were obtained; anodized brass lowered the onset potential for methanol electrooxidation (1.0 M KOH + 1.0 M CH_3_OH); based on EIS, also charge transfer resistance was low for the anodized samples	[106]

**Table 4:** Comparison of the brass electrochemical oxidation protocols.

Synthesis Conditions	Remarks
Oxidation apparatus	Two-electrode system vs. potentiostat It was found that the ordered nanostructures were applied when the potentiostat was applied (Figure 6, Figure 8 and Figure 10) [85,103,104,106]. When a two-electrode, direct-current power supply was used, an oxide with a developed surface area was obtained, rather than the well-defined nanostructures [99,100,101,102]
Type of electrolyte	Electrolytes from mildly alkaline (1.0 M NaHCO_3_ [99]) to strongly alkaline (3 M NaOH [85]) were used; there is an individual report on brass passivation in 1.0 M (COOH)_2_ [105]
Electrolyte additives	Glycerol allows for steering the Cu:Zn ratio in the grown nanostructures and tunes the morphology [104]; NH_4_Cl allows the incorporation of CuCl_2_ in the grown oxide and the formation of a more developed surface area [101]

## References

[1] Koop R., Moji Y. (1992). Boric/Sulfuric Acid Anodize-Alternative to Chromic Acid Anodize.

[2] Thompson G.E., Zhang L., Smith C.J.E., Skeldon P. (1999). Boric/Sulfuric Acid Anodizing of Aluminum Alloys 2024 and 7075: Film Growth and Corrosion Resistance. Corrosion.

[3] Thompson G.E., Furneaux R.C., Wood G.C. (1978). Electron microscopy of ion beam thinned porous anodic films formed on aluminium. Corr. Sci..

[4] Thompson R.C., Wood G.C. (1981). Porous anodic film formation on aluminium. Nature.

[5] Furneaux R.C., Rigby W.R., Davidson A.P. (1989). The formation of controlled-porosity membranes from anodically oxidized aluminium. Nature.

[6] Masuda H., Fukuda K. (1995). Ordered Metal Nanohole Arrays Made by a Two-Step Replication of Honeycomb Structures of Anodic Alumina. Science.

[7] Stępniowski W.J., Bojar Z. (2011). Synthesis of anodic aluminum oxide (AAO) at relatively high temperatures. Study of the influence of anodization conditions on the alumina structural features. Surf. Coat. Technol..

[8] Belwalkar A., Grasing E., Van Geertruyden W., Huang Z., Misiolek W.Z. (2008). Effect of processing parameters on pore structure and thickness of anodic aluminum oxide (AAO) tubular membranes. J. Memb. Sci..

[9] Kikuchi T., Onoda F., Iwai M., Suzuki R.O. (2021). Influence of sub-10 nm anodic alumina nanowire morphology formed by two-step anodizing aluminum on water wettability and slipping behavior. Appl. Surf. Sci..

[10] Ono S., Saito M., Asoh H. (2005). Self-ordering of anodic porous alumina formed in organic acid electrolytes. Electrochim. Acta.

[11] Nishinaga O., Kikuchi T., Natsui S., Suzuki R.O. (2013). Rapid fabrication of self-ordered porous alumina with 10-/sub-10-nm-scale nanostructures by selenic acid anodizing. Sci. Rep..

[12] Stępniowski W.J., Nowak-Stępniowska A., Michalska-Domańska M., Norek M., Czujko T., Bojar Z. (2014). Fabrication and geometric characterization of highly-ordered hexagonally arranged arrays of nanoporous anodic alumina. Pol. J. Chem. Technol..

[13] Nakajima D., Kikuchi T., Yoshioka T., Matsushima H., Ueda M., Suzuki R.O., Natsui S. (2019). A Superhydrophilic Aluminum Surface with Fast Water Evaporation Based on Anodic Alumina Bundle Structures via Anodizing in Pyrophosphoric Acid. Materials.

[14] Stępniowski W.J., Norek M., Michalska-Domańska M., Bojar Z. (2013). Ultra-small nanopores obtained by self-organized anodization of aluminum in oxalic acid at low voltages. Mater. Lett..

[15] Akiya S., Kikuchi T., Natsui S., Suzuki R.O. (2015). Optimum Exploration for the Self-Ordering of Anodic Porous Alumina Formed via Selenic Acid Anodizing. J. Electrochem. Soc..

[16] Stępniowski W.J., Forbot D., Norek M., Michalska-Domańska M., Król A. (2014). The impact of viscosity of the electrolyte on the formation of nanoporous anodic aluminum oxide. Electrochim. Acta.

[17] Stępniowski W.J., Moneta M., Norek M., Michalska-Domańska M., Scarpellini A., Salerno M. (2016). The influence of electrolyte composition on the growth of nanoporous anodic alumina. Electrochim. Acta.

[18] Rozenblium I., Yuferov Y., Borodianskiy K. (2024). A Comprehensive Study of Aluminum Anodization in Transition Modes. Materials.

[19] Cantelli L., Santos J.S., Trivinho-Strixino F. (2016). The effect of anodization temperature on optical properties of nanoporous anodic alumina (NAA) films. J. Electroanal. Chem..

[20] Stępniowski W.J., Zasada D., Bojar Z. (2011). First step of anodization influences the final nanopore arrangement in anodized alumina. Surf. Coat. Technol..

[21] Masuda H., Hasegwa F., Ono S. (1997). Self-ordering of cell arrangement of anodic porous alumina formed in sulfuric acid solution. J. Electrochem. Soc..

[22] Nowak-Stępniowska A. (2015). A review of quantitative arrangement analysis methods applied to nanostructured anodic oxides characterization. Curr. Nanosci..

[23] Ono S., Saito M., Ishiguro M., Asoh H. (2004). Controlling factor of self-ordering of anodic porous alumina. J. Electrochem. Soc..

[24] Nielsch K., Choi J., Schwirn K., Wehrspohn R.B., Gösele U. (2002). Self-ordering Regimes of Porous Alumina: The 10% Porosity Rule. Nano Lett..

[25] Stępniowski W.J., Michalska-Domańska M., Norek M., Czujko T. (2014). Fast Fourier transform based arrangement analysis of poorly organized alumina nanopores formed via self-organized anodization in chromic acid. Mater. Lett..

[26] Stȩpniowski W.J., Nowak-Stȩpniowska A., Presz A., Czujko T., Varin R.A. (2014). The effects of time and temperature on the arrangement of anodic aluminum oxide nanopores. Mater. Character..

[27] Norek M., Łażewski M. (2017). Manufacturing of highly ordered porous anodic alumina with conical pore shape and tunable interpore distance in the range of 550 nm to 650 nm. Mater. Sci..

[28] Norek M., Włodarski M. (2018). Morphological and chemical characterization of highly ordered conical-pore anodic alumina prepared by multistep citric acid anodizing and chemical etching process. J. Porous. Mater..

[29] Brzózka A., Szeliga D., Kurowska-Tabor E., Sulka G.D. (2016). Synthesis of copper nanocone array electrodes and its electrocatalytic properties toward hydrogen peroxide reduction. Mater. Lett..

[30] He S., Xie W., Fang S., Huang X., Zhou D., Zhang Z., Du J., Du C., Wang D. (2019). Silver films coated inverted cone-shaped nanopore array anodic aluminum oxide membranes for SERS analysis of trace molecular orientation. Appl. Surf. Sci..

[31] Ku C.-A., Yu C.-Y., Hung C.-W., Chung C.-K. (2023). Advances in the Fabrication of Nanoporous Anodic Aluminum Oxide and Its Applications to Sensors: A Review. Nanomaterials.

[32] Kim B., Lee J.S. (2016). Formation of Anodic Aluminum Oxide with Branched and Meshed Pores. J. Nanosci. Nanotechnol..

[33] Zaraska L., Kurowska E., Sulka G.D., Jaskuła M. (2012). Porous alumina membranes with branched nanopores as templates for fabrication of Y-shaped nanowire arrays. J. Solid State Electrochem..

[34] Ganapathi A., Swaminathan P., Neelakantan L. (2019). Anodic Aluminum Oxide Template Assisted Synthesis of CopperNanowires using a Galvanic Displacement Process for Electrochemical Denitrification. ACS Appl. Nano Mater..

[35] Stępniowski W.J., Moneta M., Karczewski K., Michalska-Domańska M., Czujko T., Mol J.M.C., Buijnsters J.G. (2018). Fabrication of copper nanowires via electrodeposition in anodic aluminum oxide templates formed by combined hard anodizing and electrochemical barrier layer thinning. J. Electroanal. Chem..

[36] Wang J., Pan S. (2017). Electrodeposition of vertically standing czujkAg nanoplates and nanowires on transparent conductive electrode using porous anodic aluminum oxide template. Nanotechnology.

[37] Schiavi P.G., Altimari P., Rubino A., Pagnanelli F. (2018). Electrodeposition of cobalt nanowires into alumina templates generated by one-step anodization. Electrochim. Acta.

[38] Fu J., Cherevko S., Chung C.H. (2008). Electroplating of metal nanotubes and nanowires in a high aspect-ratio nanotemplate. Electrochem. Commun..

[39] Gomez H., Riveros G., Ramirez D., Henriquez R., Schrebler R., Marotti R., Dalchiele E. (2012). Growth and characterization of ZnO nanowire arrays electrodeposited into anodic alumina templates in DMSO solution. J. Solid State Electrochem..

[40] Wu C., Shi J.B., Chen C.J., Lin J.Y. (2006). Synthesis and optical properties of ordered 30 nm PbS nanowire arrays fabricated into sulfuric anodic alumina membrane. Mater. Lett..

[41] Chen R., Xu D., Guo G., Gui L. (2002). Silver telluride nanowires prepared by dc electrodeposition in porous anodic alumina templates. J. Mater. Chem..

[42] Yan H., Zhang L., Shen J., Chen Z., Shi G., Zhang B. (2006). Synthesis, property and field-emission behaviour of amorphous polypyrrole nanowires. Nanotechnology.

[43] Sulka G.D., Hnida K., Brzózka A. (2013). pH sensors based on polypyrrole nanowire arrays. Electrochim. Acta.

[44] Lee W., Scholz R., Nielsch K., Gösele U. (2005). A Template-Based Electrochemical Method for the Synthesis of Multisegmented Metallic Nanotubes. Angew. Chem. Int. Ed..

[45] Yi Y., Lee J.K., Lee H.J., Uhm S., Nam S.C., Lee J. (2009). A single-step approach to create nanopottery structures for efficient water electrocatalysis. Electrochem. Commun..

[46] Liu L., Jia N., Zhou Q., Yan M., Jiang Z. (2007). Electrochemically fabricated nanoelectrode ensembles for glucose biosensors. Mat. Sci. Eng. C.

[47] Jung M., Kim J.H., Choi Y.W. (2018). Preparation of Anodic Aluminum Oxide Masks with Size-Controlled Pores for 2D Plasmonic Nanodot Arrays. J. Nanomater..

[48] Gan Q., Yu J., Liao Y., Huang W., Lin G., Wang J., Xu J., Li C., Chen S., Zheng J. (2022). Fabrication of ordered arrays of GeSn nanodots using anodic aluminum oxide as a template. Jap. J. Appl. Phys..

[49] Lee W.H., Han G., Kim J.O., Madhusudan P. (2025). Growth characteristic of Fe, Zn, and Zr oxide nanotubes and their novel application for phosphate recovery from wastewater. Separ. Purif. Technol..

[50] Tantray A.M., Shah M.A. (2020). Photo electrochemical ability of dense and aligned ZnO nanowire arrays fabricated through electrochemical anodization. Chem. Phys. Lett..

[51] Tantray A.M., Shah M.A. (2021). Photo electrochemical stability response of ZnO nanoflowers fabricated through single step electrochemical anodization. Chem. Pap..

[52] Wierzbicka E., Syrek K., Sulka G.D., Pisarek M., Janik-Czachor M. (2016). The effect of foil purity on morphology of anodized nanoporous ZrO_2_. Appl. Surf. Sci..

[53] Kikuchi T., Kawashima J., Natsui S., Suzuki R.O. (2017). Fabrication of porous tungsten oxide via anodizing in an ammonium nitrate/ethylene glycol/water mixture for visible light-driven photocatalyst. Appl. Surf. Sci..

[54] Fernandez-Domene R.M., Rosello-Marquez G., Sanchez-Tovar R., Cifre-Herrando M., García-Anton J. (2021). Synthesis of WO_3_ nanorods through anodization in the presence of citric acid: Formation mechanism, properties and photoelectrocatalytic performance. Surf. Coat. Technol..

[55] Cao J., Gao Z., Wang C., Muzammal H.M., Wang W., Gu Q., Dong C., Ma H., Wang Y. (2020). Morphology evolution of the anodized tin oxide film during early formation stages at relatively high constant potential. Surf. Coat. Technol..

[56] Zaraska L., Gawlak K., Gurgul K., Dziurka M., Nowak M., Gilek D., Sulka G.D. (2018). Influence of Anodizing Conditions on Generation of Internal Cracks in Anodic Porous Tin Oxide Films Grown in NaOH Electrolyte. Appl. Surf. Sci..

[57] Zaraska L., Bobruk M., Jaskuła M., Sulka G.D. (2015). Growth and Complex Characterization of Nanoporous Oxide Layers on Metallic Tin during One-step Anodic Oxidation in Oxalic Acid at Room Temperature. Appl. Surf. Sci..

[58] Wierzbicka E., Szaniawska-Białas E., Schultz T., Basilio A.O., Siemiaszko D., Ray K., Koch N., Pinna N., Polański M. (2024). Long-Term Stability of Light-Induced Ti^3+^ Defects in TiO_2_ Nanotubes for Amplified Photoelectrochemical Water Splitting. ChemSusChem.

[59] Domínguez-Jaimes L.P., Cedillo-González E.I., Luévano-Hipólito E., Acuña-Bedoya J.D., Hernández-López J.M. (2021). Degradation of primary nanoplastics by photocatalysis using different anodized TiO_2_ structures. J. Hazard. Mater..

[60] Shin S., Kim K., Choi J. (2013). Fabrication of ruthenium-doped TiO_2_ electrodes by one-step anodization for electrolysis applications. Electrochem. Commun..

[61] Mohapatra B.D., Sulka G.D. (2024). Review of Anodic Tantalum Oxide Nanostructures: From Morphological Design to Emerging Applications. ACS Appl. Nano Mater..

[62] Alias N., Hussain Z., Tan W.K., Kawamura G., Muto H., Matsuda A., Lockman Z. (2022). Photoreduction of Cr(VI) in wastewater by anodic nanoporous Nb2O5 formed at high anodizing voltage and electrolyte temperature. Environm. Sci. Poll. Res..

[63] Herath I., Davies J., Will G., Tran P.A., Velic A., Sarvghad M., Islam M., Paritala P.K., Jaggessar A., Schuetz M. (2022). Anodization of medical grade stainless steel for improved corrosion resistance and nanostructure formation targeting biomedical applications. Electrochim. Acta.

[64] Domínguez-Jaimes L.P., Arenas Vara M.Á., Cedillo-González E.I., Ruiz Valdés J.J., De Damborenea J.J., Conde Del Campo A., Rodríguez-Varela F.J., Alonso-Lemus I.L., Hernández-López J.M. (2019). Corrosion Resistance of Anodic Layers Grown on 304L Stainless Steel at Different Anodizing Times and Stirring Speeds. Coatings.

[65] Asoh H., Nakatani M., Ono S. (2016). Fabrication of thick nanoporous oxide films on stainless steel via DC anodization and subsequent biofunctionalization. Surf. Coat. Technol..

[66] Chilimoniuk P., Socha R.P., Czujko T. (2020). Nanoporous Anodic Aluminum-Iron Oxide with a Tunable Band Gap Formed on the FeAl3 Intermetallic Phase. Materials.

[67] Chilimoniuk P., Michalska-Domańska M., Czujko T. (2019). Formation of Nanoporous Mixed Aluminum-Iron Oxides by Self-Organized Anodizing of FeAl3 Intermetallic Alloy. Materials.

[68] Del Olmo R., Łazińska M., Durejko T., Antolak-Duda A., Tynkevych O., Zaraska L., Michalska-Domańska M. (2024). Anodization of cast and sintered Fe40Al alloy in etidronic acid: Morphological and semiconductive properties of the oxide films. J. Alloys Compd..

[69] Stepniowski W.J., Misiolek W.Z. (2018). Review of Fabrication Methods, Physical Properties, and Applications of Nanostructured Copper Oxides Formed via Electrochemical Oxidation. Nanomaterials.

[70] Stępniowski W.J., Wang K.K., Chandrasekar S., Paliwoda D., Nowak-Stępniowska A., Misiołek W.Z. (2020). The impact of ethylenediaminetetraacetic acid (EDTA) additive on anodization of copper in KHCO_3_–hindering Cu^2+^ re-deposition by EDTA influences morphology and composition of the nanostructures. J. Electroanal. Chem..

[71] Stępniowski W.J., Misiołek W.Z. (2020). The influence of electrolyte usage on the growth of nanostructured anodic films on copper in sodium carbonate aqueous solution. J. Electroanal. Chem..

[72] Brudzisz A., Giziński D., Wierzbicka E., Karczewski K., Tiringer U., Taheri P., Stępniowski W.J. (2021). Pom-pom-like nanowire clusters prepared by potentiostatic oxidation of copper in NH_4_HCO_3_ solution. Surf. Coat. Technol..

[73] Brudzisz A., Giziński D., Liszewska M., Wierzbicka E., Tiringer U., Taha S.A., Zając M., Orzechowska S., Jankiewicz B., Taheri P. (2023). Low-voltage anodizing of copper in sodium bicarbonate solutions. Electrochim. Acta.

[74] Stępniowski W.J., Yoo H., Choi J., Chilimoniuk P., Karczewski K., Czujko T. (2019). Investigation of oxide nanowires growth on copper via passivation in NaOH aqueous solution. Surf. Interfaces.

[75] Mah C.F., Beh K.P., Yam F.K., Hassan Z. (2016). Rapid Formation and Evolution of Anodized-Zn Nanostructures in NaHCO_3_ Solution. ECS J. Solid State Sci. Technol..

[76] Zaraska L., Mika K., Syrek K., Sulka G.D. (2017). Formation of ZnO nanowires during anodic oxidation of zinc in bicarbonate Electrolytes. J. Electroanal. Chem..

[77] Samir N., Eissa D.S., Allam N.K. (2014). Self-assembled growth of vertically aligned ZnO nanorods for light sensing applications. Mater. Lett..

[78] Allam N.K., Grimes C.A. (2011). Electrochemical fabrication of complex copper oxide nanoarchitectures via copper anodization in aqueous and non-aqueous electrolytes. Mater. Lett..

[79] Oyarzún Jerez D.P., López Teijelo M., Ramos Cervantes W., Linarez Pérez O.E., Sánchez J., Pizarro G.C., Acosta G., Flores M., Arratia-Perez R. (2017). Nanostructuring of anodic copper oxides in fluoride-containing ethylene glycol media. J. Electroanal. Chem..

[80] Gizinski D., Brudzisz A., Alzahrani M.R., Wang K.-K., Misiołek W.Z., Stępniowski W.J. (2021). Formation of CuOx Nanowires by Anodizing in Sodium Bicarbonate Solution. Crystals.

[81] Stępniowski W.J., Paliwoda D., Abrahami S.T., Michalska-Domańska M., Landskron K., Buijnsters J.G., Mol J.M.C., Terryn H., Misiołek W.Z. (2020). Nanorods grown by copper anodizing in sodium carbonate. J. Electroanal. Chem..

[82] Anantharaj S., Sugime H., Noda S. (2020). Ultrafast growth of a Cu(OH)_2_-CuO nanoneedle array on Cu foil for methanol oxidation electrocatalysis. ACS Appl. Mater. Interfaces.

[83] Anantharaj S., Sugime H., Yamaoka S., Noda S. (2021). Pushing the Limits of Rapid Anodic Growth of CuO/Cu(OH)_2_ Nanoneedles on Cu for the Methanol Oxidation Reaction: Anodization pH Is the Game Changer. ACS Appl. Energy Mater..

[84] Lee S.Y., Jung H., Kim N.-K., Oh H.-S., Min B.K., Hwang Y.J. (2018). Mixed Copper States in Anodized Cu Electrocatalyst for Stable and Selective Ethylene Production from CO_2_ Reduction. J. Am. Chem. Soc..

[85] Giziński D., Najderek M., Brudzisz A., Lee J., Choi J., Stępniowski W.J. (2024). Surface reorganization of oxide-derived Cu-M (M = Zn, Ag) bimetallic catalysts in CO_2_ electroreduction environment. Surf. Interf..

[86] Xie J.F., Huang Y.X., Li W.W., Song X.N., Xiong L., Yu H.Q. (2014). Efficient electrochemical CO_2_ reduction on a unique chrysanthemum like Cu nanoflower electrode and direct observation of carbon deposite. Electrochim. Acta.

[87] Sun M., Cheng J., Yamauchi M. (2024). Gas diffusion enhanced electrode with ultrathin superhydrophobic macropore structure for acidic CO_2_ electroreduction. Nat. Commun..

[88] Zhang Z., Dua R., Zhang L., Zhu H., Zhang H., Wang P. (2013). Carbon-Layer-Protected Cuprous Oxide Nanowire Arrays for Efficient Water Reduction. ACS Nano.

[89] Oyarzún D.P., Tello A., Sánchez J., Boulett A., Linarez Pérez O.E., Martin-Trasanco R., Pizarro G.d.C., Flores M., Zúñiga C. (2021). Exploration of Copper Oxide Nanoneedle Electrosynthesis Applied in the Degradation of Methylene Blue. Nanomaterials.

[90] Kim S.J., Choi J. (2008). Self-assembled arrays of ZnO stripes by anodization. Electrochem. Commun..

[91] Ono S., Kobayashi Y., Asoh H. (2008). Self-Organized and High Aspect Ratio Nanoporous Zinc Oxide Prepared by Anodization. ECS Trans..

[92] Zaraska L., Mika K., Hnida K.E., Gajewska M., Łojewski T., Jaskuła M., Sulka G.D. (2017). High aspect-ratio semiconducting ZnO nanowires formed by anodic oxidation of Zn foil and thermal treatment. Mater. Sci. Eng. B.

[93] Zamora-Peredo L., Ceballos-Valle A., Báez-Rodríguez A., Hernández-Torres J., García-González L., Orozco-Cruz R. (2019). Raman Spectroscopy of ZnO Nanowires Obtained by Electrochemical Anodization: Effect of Thermal Treatment, Voltage and Anodizing Time. ECS Trans..

[94] Masuda R., Kowalski D., Kitano S., Aoki Y., Nozawa T., Habazaki H. (2020). Characterization of Dark-Colored Nanoporous Anodic Films on Zinc. Coatings.

[95] Mika K., Wiercigroch E., Pisarek M., Kozieł M., Majda D., Lytvynenko A.S., Sulka G.D., Zaraska L. (2023). Nanostructured films formed on Zn during anodic oxidation in different carbonate-based electrolytes. Appl. Surf. Sci..

[96] Oksuz A.E., Yurddaskal M., Doluel E.C., Kartal U., Dikici T. (2023). Preparation and photocatalytic performances of ZnO nanostructures: Effects of anodization voltage and time. Surf. Interface Anal..

[97] Mika K., Syrek K., Uchacz T., Sulka G.D., Zaraska L. (2022). Dark nanostructured ZnO films formed by anodic oxidation as photoanodes in photoelectrochemical water splitting. Electrochim. Acta.

[98] Peng H., Wang X., Liu Z., Lei H., Cui S., Xie X., Hu Y., Ma G. (2023). Alleviating Zn Dendrites by Growth of Ultrafine ZnO Nanowire Arrays through Horizontal Anodizing for High-Capacity, Long-Life Zn Ion Capacitors. ACS Appl. Mater. Interfaces.

[99] Eissa D.S., El-Hagar S.S., Ashour E.A., Allam N.K. (2019). Electrochemical nano-patterning of brass for stable and visible light-induced photoelectrochemical water splitting. Int. J. Hydrogen Energy.

[100] Ryczek K., Kozieł M., Wiercigroch E., Małek K., Jarosz M., Sulka G.D., Zaraska L. (2020). Fast fabrication of nanostructured semiconducting oxides by anodic oxidation of brass. Mater. Sci. Semicond. Process..

[101] Nami M., Sheibani S., Rashchi F. (2021). Photocatalytic performance of coupled semiconductor ZnO–CuO nanocomposite coating prepared by a facile brass anodization process. Mater. Sci. Semicond. Process..

[102] Dezfoolian M., Rashchi F., Nekouei R.K. (2015). Synthesis of copper and zinc oxides nanostructures by brass anodization in alkaline media. Surf. Coat. Technol..

[103] Gizinski D., Mojsilovic K., Brudzisz A., Tiringer U., Vasilic R., Taheri P., Stępniowski W.J. (2022). Controlling the Morphology of Barrel-Shaped Nanostructures Grown via CuZn Electro-Oxidation. Materials.

[104] Brudzisz A., Giziński D., Lee J., Ibrahim M., Gocman K.S., Choi J., Stępniowski W.J. (2023). Electrochemical oxidation of brass in electrolytes with different viscosities. Electrochim. Acta.

[105] Al-Osta A., Jadhav V.V., Zate M.K., Mane R.S., Hui K.N., Han S.H. (2015). Electrochemical supercapacitors of anodized-brass-templated NiO nanostructured electrodes. Script. Mater..

[106] Jha S.K., Kumari A., Modalavalasa U.B.R., Singh S.K. (2024). Nanostructure-induced inhibition of oxygen evolution and enhancement of methanol electrooxidation on engineered anodized brass. Int. J. Hydrogen Energy.

[107] Juhl A.D. (2009). Why it makes sense to upgrade to pulse anodizing: Mock finishing shop scenarios show how switching from conventional anodizing processes to newer methods can boost productivity while maximizing ROI. Metal Finish..

